# The mechanism of programmed death and endoplasmic reticulum stress in pulmonary hypertension

**DOI:** 10.1038/s41420-023-01373-6

**Published:** 2023-02-25

**Authors:** Yang Sun, Shasha Liu, Chen Chen, Songwei Yang, Gang Pei, Meiyu Lin, Ting Wang, Junpeng Long, Qian Yan, Jiao Yao, Yuting Lin, Fan Yi, Lei Meng, Yong Tan, Qidi Ai, Naihong Chen, Yantao Yang

**Affiliations:** 1 Hunan Engineering Technology Center of Standardization and Function of Chinese Herbal Decoction Pieces, College of Pharmacy, Hunan University of Chinese Medicine, Changsha, P. R. China; 2 Department of Pharmacy, Changsha Hospital for Matemal & Child Health Care, Changsha, P. R. China; 3 Department of Pharmacy, The First Hospital of Lanzhou University, Lanzhou, P. R. China; 4 Department of Rehabilitation Medicine, Zhuzhou Central Hospital, Zhuzhou, P. R. China; 5 Key Laboratory of Cosmetic, China National Light Industry, Beijing Technology and Business University, Beijing, P. R. China; 6 Department of nephrology, Xiangtan Central Hospital, Xiangtan, P. R. China; 7 State Key Laboratory of Bioactive Substances and Functions of Natural Medicines Institute of Materia Medica & Neuroscience Center, Chinese Academy of Medical Sciences and Peking Union Medical College, Beijing, P. R. China

**Keywords:** Apoptosis, Necroptosis, Heart failure

## Abstract

Pulmonary hypertension (PH) was a cardiovascular disease with high morbidity and mortality. PH was a chronic disease with complicated pathogenesis and uncontrollable factors. PH was divided into five groups according to its pathogenesis and clinical manifestations. Although the treatment and diagnosis of PH has made great progress in the past ten years. However, the diagnosis and prognosis of the PAH had a great contrast, which was not conducive to the diagnosis and treatment of PH. If not treated properly, it will lead to right ventricular failure or even death. Therefore, it was necessary to explore the pathogenesis of PH. The problem we urgently need to solve was to find and develop drugs for the treatment of PH. We reviewed the PH articles in the past 10 years or so as well as systematically summarized the recent advance. We summarized the latest research on the key regulatory factors (pyroptosis, apoptosis, necroptosis, ferroptosis, and endoplasmic reticulum stress) involved in PH. To provide theoretical basis and basis for finding new therapeutic targets and research directions of PH.

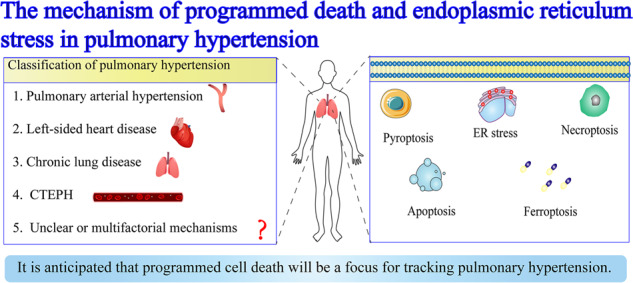

## Facts

- The incidence of endoplasmic reticulum stress is high in hypoxic pulmonary hypertension. More reasons are related to the excessive proliferation of smooth muscle cell, leading to pulmonary arteriole remodeling.
- Ferroptosis is closely related to pulmonary hypertension (PH). It may be related to the increase of active reactive oxygen species (ROS).
- Endothelial cell apoptosis contributes to vascular remodeling and triggers PH.
- PH can trigger classical and nonclassical pyroptosis.

## Open Questions

- What is the potential mechanism of endoplasmic reticulum stress and programmed cell death in pulmonary hypertension?
- How endoplasmic reticulum stress leads to pulmonary arteriolar remodeling?
- What role does programmed cell death play in different stages of pulmonary hypertension?

## Introduction

PH was a disease with high incidence rate; according to incomplete statistics, it may affect about 1% of the world’s population. Among people over 65 years old, the prevalence of pulmonary hypertension was considered to be about 10%, while 1% of young people suffered from PH [1]. In addition, PH in newborns, infants, children, adolescents and young people was a complex disease and is related to heart, lung and systemic diseases [2]. PH was a chronic progressive disease, which usually delayed the diagnosis and missed the best opportunity for treatment [3]. PH is defined by a mean pulmonary artery pressure greater than 20 mm Hg [4]. PH was a complex chronic disease, which leads to right heart failure and eventually death [5]. The pathogenesis of PH is complex and diverse, which is characterized by increased pulmonary vascular resistance [6]. PH was a chronic disease characterized by excessive pulmonary vasoconstriction and abnormal vascular remodeling [7]. Accumulating evidences showed that inflammatory response played a crucial role in the pathogenesis of PH [8]. The development of PH disease was accompanied by pulmonary fibrosis, which leads to a significant increase in morbidity and mortality [9].

Pyroptosis has attracted more and more attention because of its relationship with innate immunity and diseases. With the discovery of the gasdermin family, the research scope of cell scorch death was expanding [10]. A lot of evidence shows that the relationship between pyrosis and PH was divers. Cell death was once thought to be the result of one of two different processes, namely apoptosis (also known as programmed cell death) or necrosis (uncontrolled cell death) [11]. Programmed cell death pathways include apoptosis, pyroptosis, necroptosis, and ferroptosis [12–14]. When mitochondrial integrity was impaired byendoplasmic reticulum (ER) pressure, it will trigger the apoptosis and autophagy [15]. The cellular mechanisms involved above were interesting and have broad exploration space. Previous studies have tended to focus on tumor cells and cancer metastasis. There was very little research on PH. In this review, we summarized the relationship between PH and pyroptosis, apoptosis, necroptosis, ferroptosis, as well as ER stress.

## The overview of PH

PH is a progressive disease with complex pathogenesis, and the changes affect about 100 million people in the world [7]. PH can be clinically classified into five subgroup according to pathogenesis and clinical symptoms: (1) pulmonary arterial hypertension (PAH), (2) PH due to left-sided heart disease, (3) PH due to chronic lung disease, (4) chronic thromboembolic PH (CTEPH), (5) PH with an unclear or multifactorial mechanisms [16]. At the same time, the first group is divided into the following types: (1) Idiopathic pulmonary arterial hypertension (2) Heritable pulmonary arterial hypertension (3) Drug-induced and toxin-induced forms of pulmonary arterial hypertension (4) Pulmonary arterial hypertension associated with identified diseases (5) Persistent pulmonary hypertension of the newborn [7]. PH was defined as mean pulmonary artery pressure (mPAP)å 20 mmHg [17]. Clinical studies and animal experiments show that the pathophysiological characteristics of PAH were different degrees of perivascular inflammatory cell infiltration. The increased levels of cytokines and chemokines in pulmonary vessels leaded to excessive contraction of pulmonary vessels, which leaded to pulmonary vascular remodeling [18]. Studies have shown that inflammation is closely related to pulmonary vascular remodeling in PH. The production and accumulation of macrophages have been found in the early stage of the disease process. And T cells、mast cells、B cells、neutrophils and dendritic cells have been activated and accumulated in the pulmonary vessels of PAH patients [18, 19]. In addition, the abnormal proliferation of pulmonary vessels is related to a variety of growth factors, which can cause inflammation, proliferation and resist apoptosis, including, including platelet-derived growth factor (PDGF), basic fibroblast growth factor (b-FGF), epidermal growth factor (EGF) and vascular endothelial growth factor (VEGF) [16]. PH was a chronic disease characterized by persistent pulmonary vasoconstriction and abnormal vascular proliferation [20, 21]. In addition, studies have shown that pyroptosis、apoptosis and necroptosis contribute to PH [22, 23].

## Pyroptosis and PH

### The introduction of pyroptosis

**Fig. 1:**
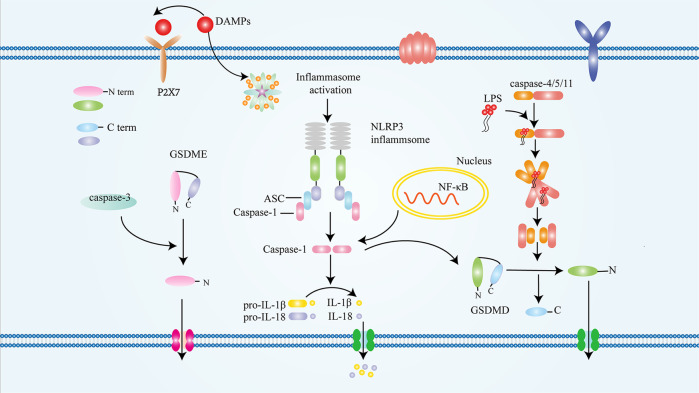
The mechanism of pyroptosis. Both traditional and nontraditional mechanisms contribute to pyroptosis.

The earliest study of pyroptosis was in 1986 [10]. The first description of molecular interactions in pyroptosis was in 1992, while the concept of pyroptosis was first proposed in 2001 [24]. NOD-, LRR- and pyrin domain-containing 3 (NLRP3) inflammasome was first found to mediate pyroptosis in 2002. Because of its caspase-dependent, nuclear concentration and DNA damage characteristics, pyroptosis was initially considered as apoptosis [10]. In addition, the most prominent feature of pyroptosis is the rapid rupture of plasma membrane and the release of pro-inflammatory contents [25]. Traditionally, caspase-1 triggered pyroptosis, but studies have found that caspase-4/5/11 and apoptosis effect caspase-3 can also induce pyroptosis [25]. In subsequent studies, caspase-8 was also shown to trigger pyroptosis [26]. In addition, it has been proposed that caspase-8 triggers pyroptosis by cutting gasdermin D (GSDMD). However, human (caspase-4/5) and mouse (caspase-11) can bind to intracellular lipopolysaccharide. Cleavage of GSDMD in turn indirectly activates IL-18 and IL-1β [26, 27]. The diameter of GSDMD-induced pore formation in cell membrane is 18 nm, and ions can flow freely in the pore [27]. When the inflammasome was activated, the N-terminal and C-terminal links between GSDMD were cleaved, and the C-terminal had an inhibitory effect on the N-terminal. After the link cleavage, the N-terminal loses its inhibitory effect, connects with phosphatidylinositol (PI) on the cell membrane, and then GSDMD channels were formed. Subsequently, a large number of inflammatory contents are released [28]. More interestingly, studies have shown that when GSDME was highly expressed, activated caspase-3 triggered pyroptosis, while less GSDME induced apoptosis [29, 30] (More detailed mechanisms are shown in the Fig. 1). In general, pyroptosis is divided into classic and non-classical pyroptosis. Interestingly, classic pyroptosis is mediated by activation of caspase-1, while most non-classical pyroptosis is closely related to caspase4/5/11, caspase-3 and caspase-8.

### Pyroptosis is involved in PH

In the model of experimental PH, western blot showed that the levels of IL-18 and caspase-1 protein in the pulmonary artery were significantly increased. Interestingly, this may contribute to pulmonary vascular remodeling in PH [31]. Previous study has shown that pyroptosis was related to hypoxia-induced PH. The results showed that the protein levels of NLRP3, ASC, caspase-1, pro-caspase-1 and IL-18 increased significantly under hypoxia. Glioma-associated oncogene family zinc finger 1 (GLI1) is a transcriptional activator. In addition, GLL was conducive to pyroptosis in hypoxia-induced PH [22]. Endothelial dysfunction was a very important pathological feature in PH, which accelerated the disease process of PH [32]. The study confirmed that the level of caspase-4/11 increased significantly in PAH animal model. In addition, caspase-4/11 can be activated in tumor necrosis factor (TNF)-α-induced human pulmonary artery endothelial cells. In addition, caspase-4 knockout inhibited the activation of GSDME and GSDMD in TNF-α-induced human pulmonary artery endothelial cells. Which suggested that pyroptosis was involved in the disease process of PAH [33]. Clinical data suggest that human high-mobility group box-1 (HMGB1) (released from pyroptosis cells) levels are elevated in patients with idiopathic or congenital heart disease-related PAH [34]. In addition, Circular RNAs (circRNAs) are a unique class of noncoding RNAs. CircRNAs mediated caspase-3 to regulate pyroptosis in pulmonary artery smooth muscle cells (PASMCs), resulting in PASMCs pyroptosis and PH [35]. The content of GSDMD increased significantly in the lungs of PH mice systemic lupus erythematosus (SLE) along with PH [36]. Although a large number of animal and clinical data have shown that both classical and non-classical pyroptosis are involved in the disease process of PH. However, there is no relevant data that can clearly show the relationship between pyroptosis and different subtypes of PH, and the underlying pathogenesis is still unclear, which is a problem we need to solve urgently.

## Apoptosis and PH

### The introduction of apoptosis

**Fig. 2:**
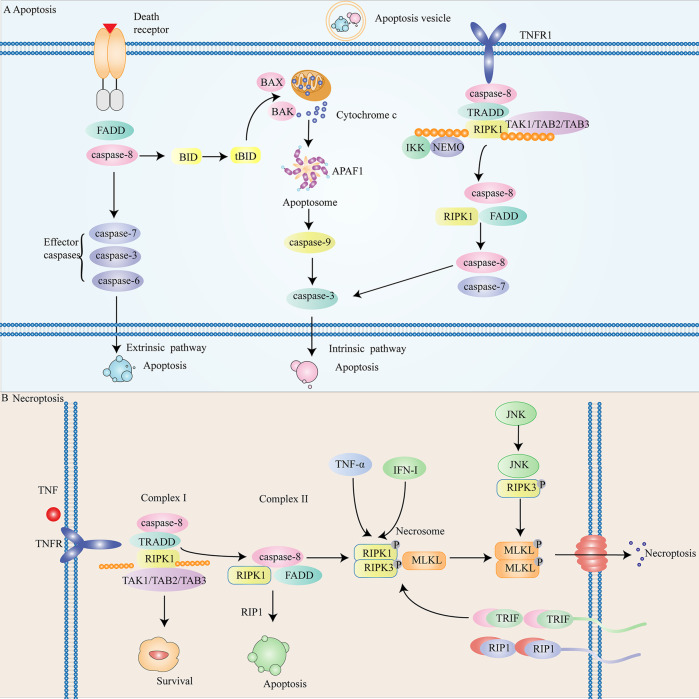
The mechanism of apoptosis and necroptosis. **A** Intracellular and extracellular routes are among the apoptotic processes. **B** The release mechanism of cells in necroptosis.

Apoptosis was a form of programmed cell death. Generally speaking, whether apoptosis occurs or not depends on the balance between apoptotic proteins and anti-apoptotic proteins [37]. Sometimes, although apoptosis in microbial infection leaded to tissue damage, activating apoptosis may be beneficial to the host because it promotes microbial clearance [38]. Apoptosis played a very important role in normal cell development, maintaining tissue homeostasis and preventing cancer [39]. Many characteristic morphological changes in cell structure and many enzyme-dependent biochemical processes were the remarkable characteristics of apoptosis [11]. The morphological characteristics of apoptosis were chromatin concentration and nuclear fragmentation, accompanied by cell aggregation, cell volume reduction and pseudopodia contraction. Meanwhile, the biochemical characteristics of apoptosis mainly included the following aspects: (1) caspase activation, (2) DNA and protein decomposition, (3) membrane changes and recognition of phagocytes [40]. Caspase was a crucial protein in apoptosis. Caspase-3, caspase-6 and, caspase-7 were effector caspases while caspase-8, caspase-9, caspase-2, caspase-10, and caspase-11 were initiator caspases [41, 42]. Proapoptotic proteins (Bax, Bak, Bok, Bid, and Bim) and anti-apoptotic (Bcl-2, Bcl-XL, and Mcl-1) proteins form Bcl-2 family. Bcl-2 protein was a key regulator of the intrinsic apoptosis pathway [41]. The interaction of these proteins constitutes the signal pathway of apoptosis, apoptosis occurs when Bcl-2 family is out of balance [37, 43]. Apoptosis was not only the most concerned form of death, but also the most studied form of cell death [39]. It was previously believed that apoptosis would not cause inflammatory response. However, this conclusion has recently been overturned. Bax/bak macropores on the mitochondrial membrane contributed to the release of mitochondrial DNA into the cytoplasm, which activated pro-inflammatory signals in the absence of capsase. In fact, apoptosis was a positive and meaningful physiological process. In many organisms, apoptosis was a normal and universal physiological process [39, 42] (More detailed mechanisms are shown in the Fig. 2A). In general, apoptosis is the most common form of programmed cell death and has very important physiological functions (maintaining tissue homeostasis and preventing cancer). In addition, apoptosis is closely related to multiple signal pathways and signal transduction. Therefore, apoptosis is also receiving more and more attention.

### Apoptosis is involved in PH

Studies have shown that the apoptosis of pulmonary artery endothelial cells is reduced, and the expression of Bcl-2 proteins is significantly increased while Mcl-1 decreased in IPAH [44]. In the right ventricle of SU5416/hypoxia-treated rats, it was found that p53 increased significantly, and p53 promoted right ventricular apoptosis [45]. TUNEL staining showed that endothelial cell apoptosis was observed in CTEPH model rats. In addition, the results of western blot and immunohistochemistry showed that the content of Bcl-2 protein increased significantly in rat pulmonary vessels [46]. Studies have shown that endothelial cell apoptosis is a driving factor in the induction of PAH [47]. Precapillary pulmonary artery stenosis and occlusion are prominent features of IPAH, which secondary to proliferation and apoptosis resistance of endothelial cells, smooth muscle cells, and fibroblasts [48]. TGF-β1 induced PASMCs excessive proliferation and reduced apoptosis through Smad2/3, so as to induce PH [49]. In addition, the main reason for vascular remodeling is the balance between apoptosis and the proliferation of endothelial cells and smooth muscle cells. Studies have confirmed that under hypoxic conditions, promoting smooth muscle cell apoptosis effectively improved vascular remodeling [50]. There are many possible mechanisms for endothelial cell apoptosis to participate in PAH. For example, endothelial cell apoptosis leads to cell abscission, which increases pulmonary vascular resistance; Apoptotic endothelial cells lose their inhibitory effect on the proliferation of smooth muscle cells, leading to vascular remodeling. Therefore, endothelial cell apoptosis plays an important role in PAH [46]. Studies have shown that decreased endothelial apoptosis improves cardiac and arterial remodeling in PAH [51]. Studies have shown that early apoptosis contributes to the increase of vascular cells, and some researchers even believed that endothelial cell apoptosis is the direct cause of PH. Therefore, there is a close relationship between endothelial cell apoptosis and pulmonary vascular remodeling [52, 53]. Endothelial cells and smooth muscle cells were mostly studied in PH. Endothelial cell apoptosis caused endothelial dysfunction or apoptosis caused by endothelial dysfunction, which eventually leads to PH. The specific reason was not clear. Although there is a close relationship between apoptosis and PH in animal and cell-level studies, more clinical data are needed to support this conclusion.

## Necroptosis and PH

### The introduction of necroptosis

Necroptosis was initially considered as accidental and uncontrolled cell death. With the deepening of research, researchers found that necroptosis was also regular [54]. Necroptosis was similar to apoptosis in both mechanism and morphology. Necroptosis was a form of programmed death and was regulated by members of the tumor necrosis factor receptor (TNFR) superfamily, pattern recognition receptors (PRRs), T cell receptors (TCRs) as well as multiple chemotherapeutic drugs [55]. In addition, cytosolic nucleic acid sensors induced type I interferon (IFN-I) and TNF-α production and thus promoted necroptosis in an autocrine feedback loop [56]. The activation (including ubiquitination and phosphorylation) of receptor-interacting protein kinase1 (RIPK1), receptor-interacting protein kinase 3 (RIPK3) and mixed lineage kinase domain-like protein (MLKL) were involve in the key regulatory mechanisms [57]. The results showed that RIPK1, RIPK3 and MLKL were not found in primitive organisms. RIPK1 was present in most vertebrate species and mammals while RIPK3 and MLKL exist only in some vertebrates and mammals [58]. RIPK3 interactd with RIPK1 kinase and regulated its activity. In the context of RIPK31-RIPK1 complex (necrotic body), RIPK1 activated RIPK3, which in turn activated MLKLto trigger necroptosis [59]. RIPK was found to be the first genetic determinant of necroptosis induced by death receptor (DR). Necroptosis usually occured when the apoptotic signal component of DR losed its function [24]. The activation of TNF signaling pathway was the driving factor of necroptosis. The combination of TNF and TNFR1 induces the change of TNFR1 trimer, resulting in the recruitment and recruitment of a variety of proteins, including PIPK1, TRADD (TNFR related death domain), CIAPI (apoptosis protein 1 inhibitor), CIAP2, TRAF2 (TNFR related factor 2) and TRAF5 [55]. Studies have shown that mutant mice that lose the key components of apoptosis mechanism (CASP3 −/− mice and CASP9 −/− mice) show serious developmental defects. On the contrary, mutant mice that lacked the key components of necrotic apoptosis mechanism (RIPK3 −/− and MLKL −/− mice) or lacked the kinase activity of RIPK1, which showed normal development [58] (More detailed mechanisms are shown in the Fig. 2B). Although necroptosis is also a form of programmed cell death, it is obviously not as common as apoptosis.

### Necroptosis is involved in PH

Xiao et al. confirmed the increased expression of RIPK3, MLK and RIPK1 after 4 weeks of monocrotaline (MCT) treatment by PCR. These results suggest that RIPK3-mediated necrotic apoptosis was enhanced in MCT-induced PAH [60]. Studies have shown that reducing necrosis rather than necroptosis reduced TLR4 signal transduction in PAH male rats [61]. Although necroptosis was rarely studied in PH, it has been found that mitochondrial oxidative phosphorylation (OXPHOS) activated necroptosis in human lung epithelial cells [62]. By constructing RIPK3 adenovirus vector, some studies further confirmed that the increase of RIPK3 expression induced by JNK activation was the cause of pulmonary fibrosis in mice [63]. There were few studies on the pathogenesis of necroptosis in PH. Indeed, studies have confirmed that necroptosis contributes to the development of PH disease, but the relevant mechanism and key regulatory molecules were not clear.

## Ferroptosis

### The introduction of ferroptosis

**Fig. 3:**
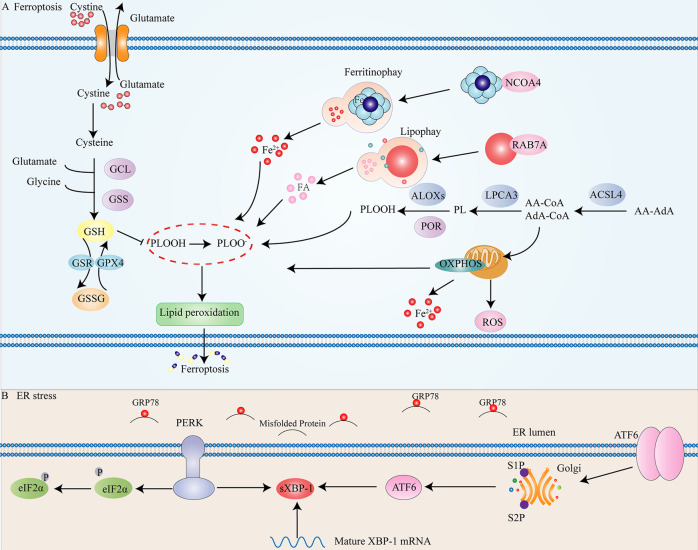
The mechanism of ferroptosis and endoplasmic reticulum stress. **A** Release process of ferroptosis cells. **B** Activation of sXBP-1 in ER stress.

Ferroptosis is a term put forward in 2012, and iron death has become a research hotspot in recent years. This unique cell death mode driven by iron-dependent phospholipid peroxidation is regulated by a variety of cellular metabolic events, including redox homeostasis, iron handling, mitochondrial activity and metabolism of amino acids, lipids and sugars, as well as numerous disease-related signaling pathways [64]. Ferroptosis is an iron dependent, nonapoptotic regulatory cell death caused by lipid peroxidation, which is controlled by a comprehensive oxidation and antioxidant system. Iron-containing enzyme lipoxygenase is the main promoter of iron death. Its function depends on the activation of ACSL4-dependent lipid biosynthesis through the production of lipid hydroperoxide [65]. With the continuous deepening of research, the research shows that the mechanism of inducing and inhibiting iron death is more diversified than originally thought, which is beyond the glutathione peroxidase 4 (GPX4) axis [66]. Ferroptosis is an iron and reactive oxygen species (ROS) dependent cell death characterized by cytological changes, including the reduction or disappearance of mitochondrial cristae, mitochondrial outer membrane rupture and mitochondrial membrane concentration. The reason for the above cytological changes is that the occurrence of strong membrane lipid peroxidation (LPO) and oxidative stress leads to the loss of selective permeability of the plasma membrane [67]. In addition, in some cases, ferroptosis is accompanied by the separation and aggregation of cells and the increase of autophages [68]. Studies have shown that iron death-inducing factors can directly or indirectly affect GPX4 through different ways, leading to the decline of intracellular antioxidant capacity and the accumulation of lipid ROS, and ultimately leading to oxidative cell death [69]. Two key initial signals have been proposed to trigger iron death: excessive iron accumulation and inhibition of GPX4 [70]. Research shows that the basis of ferroptosis is the accumulation of iron and LPO in cells [71]. Ferroptosis can be prevented by the enzymatic reaction of two major antioxidant systems, including GPX4, which catalyzes the reduction of lipid peroxides in glutathione-dependent reactions, and the recently discovered iron death inhibitory protein (FSP1) that promotes the regeneration of ubiquinone [72]. Ferroptosis can be induced by many compounds and drugs and regulated by many proteins and genes. Ferroptosis also involves multiple metabolic pathways, including iron metabolism, amino acid and lipid metabolism [73]. It is believed that ferroptosis is the main driving factor of nerve cell death in Parkinson’s disease (PD) and Alzheimer’s disease (AD). Epidemiology and animal studies show that the iron-rich microenvironment characterized by malignant tumors supports rapid proliferation and contributes to carcinogenesis [74]. In addition, ferroptosis is closely related to a variety of cardiovascular diseases, including cardiomyopathy, myocardial infarction, ischemia/reperfusion injury, and heart failure [75] (More detailed mechanisms are shown in the Fig. 3A). Obviously, ferroptosis has attracted more and more attention. The possible reason is that ferroptosis is closely related to the pathogenesis of many diseases.

### Ferroptosis is involved in PH

Xie et al. detected the relative levels of iron death markers (GPX4, ferritin heavy chain 1 (FTH1) and oxidase 4 (NOX4)) and Fe^2+^ in lung samples by western blotting. Compared with sham-operated rats, the expression levels of GPX4 and FTH1 proteins in rats induced by MCT decreased and the expression of NOX4 increased. In addition, iron determination showed that the level of Fe^2+^ in rats induced by MCT increased. Ferroptosis inhibitor ferrostatin-1 (Fer-1) treatment inhibited these changes in MCT-induced rats, but not in the sham+Fer-1 group. In addition, the study showed that ferroptosis cells released high-mobility group box 1(HMGB1), which was almost absent in the supernatant of cell culture in the blank group, while HMGB1 in the model group was significantly enhanced. However, when ferroptosis is inhibited by Fer-1, HMGB1 levels are inhibited. In addition, xie et al. further studied whether TLR4 is involved in the activation of NLRP3 inflammatory bodies in macrophages. The results showed that HMGB1 treatment increased the macrophage expression of TLR4 and NLRP3 inflammatory markers (NLRP3, ASC, pro-caspase-1, caspase-1, and the ratio of caspase-1/pro-caspase-1) and inflammatory cytokines (IL-1β、IL-18 and TNF-α). While the TLR4 antagonist TAK-242 has the opposite effect. Ferroptosis regulates PH through HMGB1/TLR4/NLRP3 inflammatory signaling pathway [76]. In addition to highlighting systemic iron deficiency, intracellular iron deficiency also plays a crucial role in the pathogenesis of PAH. The study shows that rats fed iron deficiency diet have similar pulmonary vascular remodeling and hemodynamic changes as those of PAH patients, which can be reversed by iron supplementation [77]. Ferroptosis-related genes are mainly involved in oxidative stress and can be regulated by several identified microRNA (miRNA) and gene-transcription factor (TF). This suggests that there are regulatory pathways that may be involved in PAH-related ferroptosis [78]. The research shows that erastin induced ferroptosis by inhibiting the expression of solute carrier family 7 member 11 (SLC7A11) and glutathione peroxidase 4 (GPX4) in vivo and in vitro, which indicates that erastins reverses the continuous proliferation of hypoxic PASMCs [79]. Although research shows that ferroptosis is closely related to PH. However, there is little research on the pathogenesis of ferroptosis in PH. More may be related to the increase of active ROS.

## Endoplasmic reticulum stress and PH

### The introduction of endoplasmic reticulum stress

Endoplasmic reticulum (ER) was the central organelle responsible for the synthesis, folding and modification of secreted and transmembrane proteins [80]. ER was the largest and dynamic organelle in eukaryotic cells, which is responsible for the synthesis, folding and modification of more than one-third of proteins in cells [81, 82]. Generally speaking, ER stress occurs in the state of high glucose, hypoxia, oxidative stress and lack of energy [83]. ER stress refers to the accumulation of misfolded proteins in the endoplasmic reticulum. Only properly folded proteins entered the exit of the ER and leaved the ER. When the synthetic demand was excessive or the synthetic conditions did not meet the demand, misfolded or unfolded proteins accumulated, and endoplasmic ER occured. Therefore, we judged the occurrence of ER stress by whether the unfolded protein response was activated [84]. Studies have shown that interrupting protein biosynthesis, such as abnormal increase of protein biosynthesis, inhibition of disulfide bond formation, consumption of metabolic energy and disturbance of N-glycosylation, can lead to protein misfolding [85]. In the central protein involved in ER stress, the central protein was activated transcription factor 6 (ATF6) and inositol-requiring enzyme-1α (IRE1α) and protein kinase R-like endoplasmic reticulum kinase (PERK) in mammalian cells [86]. When stress was generated, the accumulation of unfolded or misfolded proteins enhanceed the release and activation of GRP78 and ER transmembrane receptor proteins [87]. Studies have shown that PERK and ATF6 signaling pathways up-regulate the expression of sXBP1 mRNA, resulting in effective transcription factors responsible for inducing the expression of stress response genes [88]. When ER stress occured, ATF6 transfered from ER to Golgi, where it was cleaved by proteases at site 1 and site 2. Free ATF6 transcription up-regulated ER chaperone protein, enhanced ER folding ability, and then participated in lipid biosynthesis [88]. Lack of PERK or phosphorylates eukaryotic initiation factor 2α (peIF2α) cells may increase the accumulation of misfolded or unfolded proteins in the ER cavity. Studies have shown that hyperglycemia for more than 18 h activated PERK, resulting in ER stress. In addition, exposure to lower glucose levels and shorter periods of time may promote PERK activation, resulting in eIF2α phosphorylation [89] (Fig. 3B). In general, ER stress refers to the accumulation of misfolded proteins in the ER. We also judge the occurrence of ER stress by whether the unfolded protein reaction is activated. But in the final analysis, ER stress is closely related to high glucose, hypoxia, oxidative stress and lack of energy.

### Endoplasmic reticulum stress is involved in PH

Studies have shown that abnormal proliferation of PASMCs promoted vascular remodeling in PH. ER stress promoted the proliferation and growth of PASMCs [83]. The researchers found that the ER markers GRP78, ATF6 and XBP1 increased in the lung homogenate of PH rats. The results showed that the pathogenesis of PAH was related to ER stress and UPR in ECs [83, 90]. Nogo was a member of the reticular protein family and was essential for regulating the tubular structure of ER. Nogo existed in three subtypes: A, B and C. Nogo-A was expressed in the central nervous system, Nogo-B was generally expressed, and Nogo was expressed in neurons and skeletal muscle. When endoplasmic reticulum stress occured, the presence of a certain level of Nogo-B may lead to ER recombination and the destruction of mitochondrial ER units, which leaded to mitochondrial hyperpolarization, the closure of mitochondrial transition pores and the inhibition of apoptosis. It showed that Nogo-B was very important for the function of mitochondrial ER unit in pulmonary circulation [91]. In addition to hypoxia, ER stress may also be caused by many different conditions leading to PAH, including the unfolded protein response of mutant BMPRII in familial PAH. In addition, the overexpression of Notch3 is related to the pathogenesis of PAH and ER stress [92]. Significant ultrastructural changes of ER in pulmonary aortic smooth muscle cells were observed by projection electron microscopy. It showed that various pathological results were related to ER stress. The researchers also founded that mitochondrial rupture in PASMC enhanced ER stress in hypoxic rats, leading to PASMC dysfunction [93]. By inhibiting IRE1α/XBP1 signaling pathway further downregulates PCNA and MMP9 and inhibits hypoxia-induced cell proliferation, migration and apoptosis [94]. In addition, ER stress not only promoted the proliferation of SMCs, but also accelerated the process of vascular remodeling by promoting inflammatory response in PH [95]. Although many studies have proved that endoplasmic reticulum stress is accompanied by PH, few studies have reported specific targets and potential molecular mechanisms. More reasons are related to the excessive proliferation of SMCs, leading to pulmonary arteriole remodeling. And ER stress occured more in hypoxia-induced pulmonary hypertension The exact mechanism needed to be further studied. ER stress was expected to become a potential target for the treatment of PH and improving ER stress may become a new treatment policy and means for PH in the future.

### Possible cross-talk of pyroptosis, apoptosis, necroptosis, ferroptosis and endoplasmic reticulum stress in PH

Pyroptosis, apoptosis, and necroptosis have long been considered as forms of programmed cell death [96, 97]. For a long time, cell death pathways have been considered to play an independent role with little interaction. However, it has been clear that apoptosis, necroptosis and pyroptosis were closely related and regulated each other [56]. Caspase-8 is the central link regulating the role of the pyroptosis, apoptosis and necroptosis. Caspase-8 not only regulated apoptosis, but also prevented the formation of necrotic bodies [98, 99]. Studies have shown that pro-caspase-8 inhibited the formation of ASC in embryonic intestinal tissue, thus promoting caspase-1-dependent GSDMD and pyroptosis [26]. There was indeed a close cross-regulation between apoptosis, necroptosis and pyroptosis. If one pathway was damaged, the relationship between these pathways will also be affected [56]. Pyroptosis, apoptosis, necroptosis, and ferroptosis were all involved in the disease process of PH. Studies have shown that ER stress-regulated pyroptosis in spinal cord ischemia-reperfusion injury [100] and cerebral venous sinus thrombosis [101]. ER stress-induced apoptosis in traumatic brain injury [102], in human renal cortex proximal tubule epithelial (HK-2) cells [103], in cardiomyocyte [104], and in cerebral ischemia reperfusion [105]. However, there were few studies on the interaction between ER stress and programmed cell death (pyroptosis, apoptosis, necroptosis, and, ferroptosis) in PH. Future research can be targeted to study the crosstalk between programmed cell death and ER stress in PH.

## Conclusion and perspective

The pathogenesis of PH was complex and unknown. Although there were many therapeutic drugs in clinic, the curative effect is poor. Therefore, it was very important to find new therapeutic targets. In this review, we summarized how apoptosis, pyroptosis, necroptosis and ER stress affect PH based on current literature. Some mechanisms need to be further clarified and studied, especially the pathogenesis of cell pyroptosis in PH. There was little research in this field. Although many studies have reported that ER stress played a role in the process of PH. However, the specific mechanism of action was still not explained clearly. At present, it can be concluded that ER stress promoted the proliferation of SMCs and eventually leaded to pulmonary arteriole remodeling. However, the specific target and specific signal pathway are not clear. This is a worthy research direction in the future.

## Data Availability

All data generated in the current study are available.
